# Case of an Extraneous Substance Lodged within the Vagina during Four Years

**Published:** 1803-12-01

**Authors:** W. Blair

**Affiliations:** Surgeon of the Lock Hospital, &c.


*Case of an extraneous Substance lodged within the Vagina during four Years; by* W. Blair, *Surgeon of the Lock Hospital, &c.*

I Was this day requested by my colleague, Dr. Pinckard, to visit a woman who had lately applied to him at the Bloomsbury Dispensary; and, if possible, to extract from her vagina a large piece of wood, which she had introduced *four years ago,* for the purpose of preventing a prolapsus uteri. On examining the patient, I found that this piece of wood had partly made its way into the rectum, so as to occasion the fæces to evacuate themselves by the vagina and external organs of generation. The smell arising from this mass of corruption was the most abominable I ever experienced; the urine and fæces mingled with the usual discharge from a gangrenous part, in a subject who by nature was the most filthy and negligent possible, produced a nauseating odour which is better imagined than described.

There would have been no difficulty, I suppose, in extracting this substance before inflammation and swelling had taken place; but as the woman had stupidly concealed her real situation till the last extremity of suffering, and had herself made ineffectual efforts to withdraw the wooden pessary, (if it may be so named) I found a little difficulty in getting it out with perfect safety; that is, without lacerating the perinæum. When it was removed, it appeared to be a quadrangular portion of coarse deal, three inches long, two in breadth, and one in thickness. What may be the result of this case, cannot now be foretold; but I fear the woman will languish only a short time, and finally sink under the symptoms of irritation, &c. with which she is afflicted.

This woman is about fifty-one years of age; about twelve months ago she was under the care of Dr. P. being afflicted with anasarca. She was then cured; but, during the last three months, she has suffered from a return of the same complaint. For a long time past she has not been able to sit down, but has been in the habit of resting upon her knees.

*Great Russel Street, Oct.* 15, 1803.

